# Short-Chain Fatty Acids Regulate the Immune Responses via G Protein-Coupled Receptor 41 in Bovine Rumen Epithelial Cells

**DOI:** 10.3389/fimmu.2019.02042

**Published:** 2019-08-28

**Authors:** Kang Zhan, Xiaoxiao Gong, Yinyin Chen, Maocheng Jiang, Tianyu Yang, Guoqi Zhao

**Affiliations:** Institute of Animal Culture Collection and Application, College of Animal Science and Technology, Yangzhou University, Yangzhou, China

**Keywords:** bovine rumen epithelial cells, immortalization, short-chain fatty acids, GPR41, immune responses

## Abstract

The rumen immune system often suffers when challenging antigens from lysis of dead microbiota cells in the rumen. However, the rumen epithelium innate immune system can actively respond to the infection. Previous studies have demonstrated G protein-coupled receptors 41 (GPR41) as receptors for short chain fatty acids (SCFAs) in human. We hypothesized that SCFAs, the most abundant microbial metabolites in rumen, may regulate the immune responses by GPR41 in bovine rumen epithelial cells (BRECs). Therefore, the objective of study was to firstly establish an immortal BRECs line and investigate the regulatory effects of SCFAs and GPR41 on innate immunity responses in BRECs. These results showed that long-term BRECs cultures were established by SV40T-induced immortalization. The concentrations of 20 mM SCFAs significantly enhanced the levels of GPR41, IL1β, TNFα, chemokines, and immune barrier genes by transcriptome analysis. Consistent with transcriptome results, the expression of GPR41, IL1β, TNFα, and chemokines were markedly upregulated in BRECs treated with 20 mM SCFAs by qRT-PCR compared with control BRECs. Remarkably, the GPR41 knockdown (GPR41KD) BRECs treated with 20 mM SCFAs significantly enhanced the proinflammatory cytokines IL1β and TNFα expression compared with wild type BRECs treated with 20 mM SCFAs, but reduced the expression of CCL20, CXCL2, CXCL3, CXCL5, CXCL8, CXCL14, Occludin, and ZO-1. Moreover, GPR41 mRNA expression is positively correlated with CCL20, CXCL2, CXCL3, CXCL8, CXCL14, and ZO-1. These findings revealed that SCFAs regulate GPR41-mediated levels of genes involved in immune cell recruitment and epithelial immune barrier and thereby mediate protective innate immunity in BRECs.

## Introduction

The bovine rumen epithelium often struggles to challenge antigens from lysis of dead microorganism cells within the rumen (1–3), but innate immune system can actively respond to the infection by free LPS from the shedding gram-negative bacteria or the lysis of dead bacterial cells in rumen. However, the factors and molecular mechanisms that regulate the innate immune response in rumen epithelium remains unknown. The rumen contains a large amount of the ruminal microbiota, which can metabolize the dietary carbohydrate into short-chain fatty acids (SCFAs) involved in the immune regulation (4–6).

SCFAs, including acetate, propionate, and butyrate, are not only major sources of daily energy requirement in ruminant animals (7, 8), but also regulate the rumen epithelium growth, leptin level, insulin secretion, and immune response processes (9–12). SCFAs are transported into the rumen epithelial cells through lipophilic diffusion or protons across the basolateral membrane via monocarboxylate transporter 1 (MCT1) pathway (13, 14). Intraruminal administration of SCFAs stimulates the rumen papilla development in young ruminants (11). In addition, SCFAs contribute to the arterial insulin concentration and glucagon concentrations (10, 15). SCFAs can also stimulate the activation of sympathetic nervous system to promote body energy expenditure (16) and reduce the fat synthesis (8). However, little information is known about the molecular mechanisms that SCFAs use to regulate the ruminal epithelium innate immune responses. We hypothesized that SCFAs may regulate the innate immune responses in BRECs.

In present study, cultures of long-term bovine rumen epithelial cells (BRECs) were established by SV40T-induced immortalization. To identify the regulatory effects of SCFAs on innate immunity responses in BRECs, we firstly found the difference between mRNA in BRECs with or without SCFAs using transcriptome analysis. Interestingly, many genes involved in chemokines and the function of immune barrier were significantly upregulated in BRECs induced by SCFAs. Past studies have demonstrated G protein-coupled receptors 43 (GPR43) and 41 (GPR41) as receptors for SCFAs in human (17–19). The GPR41 and GPR43 are differentially expressed in other types of cells, including the adipocytes, neutrophil, and colonic epithelial cells, and regulate the host energy balance and immune responses (5, 6, 9). In addition, expression of GPR41 and GPR43 mRNA can be detected in rumen epithelium, but expression of GPR43 exhibited lower level (20). Remarkably, GPR41 was persistently expressed in the immortalized BRECs, but expression of GPR43 was not observed in passage 2 of primary BRECs and immortalized BRECs. We hypothesized that SCFAs may regulate the innate immune and the function of immune barrier via GPR41 in BRECs. Therefore, the objective of study was to provide the evidence that SCFAs regulate GPR41-mediated the expression of genes involved in the diverse chemokine and immune barrier and thereby mediate protective innate immunity responses in BRECs.

## Materials and Methods

### Isolation and Cultivation of Primary Bovine Rumen Epithelial Cells

Bovine used in this study was complied with the guidelines of the Institutional Animal Care and Use Committee (IACUC) of Yang Zhou University. The abdominal sac tissues (depending on papillae density) of the rumen from three young Holstein calves were obtained from the Experimental Farm of Yang Zhou University. These Holstein calves were 6- to 7-mo-old (206.2 ± 15.3 kg) and were fed a forage ration and concentrate supplement to meet NRC requirements. Primary BRECs were obtained as described previously with minor adaptations (21). The rumen tissues were quickly excised and repeatedly rinsed using PBS containing 500 U/mL penicillin, 500 μg/mL streptomycin, 250 μg/mL gentamicin, and 12.5 μg/mL amphotericin B (5×PSGA; Invitrogen, Shanghai, China). Then, the rumen epithelium tissues were separated using blunt dissection and transported to a laboratory in a DMEM medium containing 5×PSGA on ice immediately. These tissue pieces were repeatedly washed with DMEM medium (Invitrogen) containing 5×PSGA until the supernatant was clear. Subsequently, these tissues were minced into ~1 mm^3^ size pieces, and repeatedly washed by centrifugation at 200 × g at 4°C for 1 min until the supernatant was clear. Minced epithelium was placed into the digestion flask containing 30 mL of 0.25% trypsin-0.02% EDTA. The flask was placed in a slow shaking, hot air incubator for 10 min at 37°C. The process was performed three times, and the digestion solution was discarded. These remaining epithelium tissues were digested again as described above. The process was performed four times, and the digestion solution was filtered through 74 μm nylon mesh into 50 mL sterile tube containing 10% FBS DMEM medium, and collected by centrifugation at 200 × g at 4°C for 5 min and discard the supernatant. Cells were washed with DMEM medium containing 5×PSGA for three times. Cell pellets were resuspend in the DMEM medium containing 10% FBS, 100 U/mL penicillin, 100 μg/mL streptomycin, 1% non-essential amino-acids (NEAA), 4 mm/L glutamine, 1×Insulin-Transferrin-Selenium (1×ITS; Invitrogen), 15 ng/mL EGF (peprotech, Shanghai, China), and seeded in 6-well plate. Complete attachment may require up to 72 h. Therefore, we must be careful and avoid shaking the plate.

### Bovine Rumen Epithelial Cells Immortalization and Clone

Primary BRECs were transduced with lentiviruses expressing SV40 large T antigen containing 8 μg/mL polybrene (Sigma-Aldrich) overnight. Subsequently, the viral supernatant was carefully removed, and these cells were further cultured in medium. After 8 d cultures, cells were detected using 0.05% trypsin-0.02% EDTA, and cells were diluted to 10 cells/mL using DMEM/F12 medium. Then, 200 μL cell suspension were aliquoted into each well of 96-well plates, and cells were observed every 5 days. If cells with epithelial-like morphology reached 50% confluence, and cells were transferred into 24-well plates for further culture. In this way, five immortal BRECs clones were successfully established. The immortal BRECs were collected at the Institute of Animal Culture Collection and Application (IACCA), Yangzhou University.

### Identification of Immortalized Bovine Rumen Epithelial Cells

The SV40T band was performed by Western blot. To validate the origin of immortal BRECs, reverse-transcription PCR (RT-PCR) was performed to assess the expression of genes involved in SCFAs transporters including MCT1, MCT4, Na^+^/H^+^ exchanger 1 (NHE1), NHE2, NHE3, and GPR41. The immortal BRECs with 40 mM SCFAs stimulation were collected to extract total RNA. RT-PCR was performed with an RT Kit (Takara, Beijing, China) according to the manufacturer's protocol. The PCR reaction was performed for an initial denaturation at 95°C for 30 s, followed by 40 cycles at 95°C for 5 s and 60°C for 30 s. Each reaction was performed in triplicate, using GAPDH as the internal control. PCR products were separated using 2% agarose gel electrophoresis and visualized after goldview staining.

### Proliferative Activity Analysis

Cell proliferation assays were performed using the Cell Counting Kit-8 (CCK-8; Dojindo, Shanghai, China) according to the manufacturer's protocol. 5 × 10^2^ cells/well was seeded into 96-well tissue culture plates. After incubation, 10 μL CCK-8 reagent was added, and cells were then incubated for 2 h. To determine the activity of cell proliferation, absorbance was measured for each well at a wavelength of 450 nm, using an auto-microplate reader (Thermo Scientific, Shanghai, China).

### Illumina HiSeq mRNA Sequencing

The immortalized BRECs were seeded in six-well plates (2 × 10^5^ cells/well) for transcriptome analysis. The BRECs in the absence of 20 mM SCFAs and BRECs treated by 20 mM SCFAs were cultured in DMEM/F12 medium at 37°C, 5% CO_2_ for 24 h, respectively. Total RNA was extracted from BRECs using the TRIzol reagent (Invitrogen, Shanghai, China). The RNA (1 μg) of each sample with an RNA integrity number (RIN) value above 7 was used for subsequent library preparation by Genewiz (Genewiz, Suzhou, China). Poly (A) mRNA isolation was performed using the NEB Next Poly (A) mRNA Magnetic Isolation Module (NEB, Beijing, China). The mRNA fragmentation and priming were performed using the NEB Next First Strand Synthesis Reaction Buffer and NEB Next Random Primers. First strand cDNA was synthesized using ProtoScript II Reverse Transcriptase (NEB, Beijing, China) and the second-strand cDNA was synthesized using Second Strand Synthesis Enzyme Mix (NEB, Beijing, China). The purified double-stranded cDNA was then treated with End Prep Enzyme Mix to repair both ends and add a dA-tailing in one reaction, followed by a T-Aligation to add adaptors to both ends. Size selection of adaptor-ligated DNA was then performed using AxyPrep Mag PCR Clean-up (Axygen, Shanghai, China), and fragments of ~360 bp were recovered. Each sample was then amplified by 11 cycles of PCR using P5 and P7 primers. Both primers carried sequences that were able to anneal with flow cells to achieve bridge amplification, and the P7 primer carried a six-base index that facilitated multiplexing. PCR products were cleaned up using AxyPrep Mag PCR Clean-up (Axygen, Shanghai, China), validated using an Agilent 2100 Bioanalyzer (Agilent Technologies, Palo Alto, CA, USA), and quantified with a Qubit 2.0 Fluorometer (Invitrogen, Shanghai, China). Then libraries with different indices were multiplexed and loaded on an Illumina Hiseq×10 instrument according to manufacturer's instructions (Illumina, San Diego, CA, USA). Sequencing was carried out using a 2×150 bp paired-end (PE) configuration and obtained the ~8.5 G raw data bases. Kyoto Encyclopedia of Genes and Genomes (KEGG) pathway analysis was used to determine the significant pathways associated with the differentially expressed genes identified.

### Generation of GPR41 Knockdown BRECs

The GPR41 targeted gRNA expression oligos were introduced into the pGK1.1 vector (Genloci, Nanjing, China). The sequences of these oligos are shown in **Figure 3A**. A mixture of 6 μg of pGK1.1 plasmid DNA containing each target gRNA sequence was electrotransfected into BRECs (5 × 10^6^ cells). The transfected cells were cultured for 3 days. Then, cells were isolated using 0.05% trypsin-0.02% EDTA, and cells were diluted to aliquot into each well of 10 96-well plates. Single cell colony was select to extract the genome DNA. The genome DNA was isolated using a TNA Kit (Genloci). The genomic region surrounding the CRISPR/Cas9 target gRNA site was amplified using the GPR41 primer (**Figure 3A**). The amplicons were incubated using Cruiser™ Enzyme (Genloci) to screen the positive target. Amplicons digested by Cruiser™ Enzyme were separated in 2% agarose gel electrophoresis. The positive clone was performed the sequencing analysis. Finally, the amplicon sequences were cloned into T vector (Takara, Beijing, China). A colony was selected and the sequencing analysis was performed to determine the knockout target site.

### Culture of BRECs

The passages 3 immortalized BRECs were seeded in six-well plates (2 × 10^5^ cells/well) for mRNA expression analysis. Cells were divided into three experimental groups in the following manner: wild type BRECs were cultured in pH 7.2 DMEM/F12 medium in the absence (control groups) or presence of 20 mM SCFAs, or GPR41 knockdown (GPR41KD) BRECs were cultured in pH 7.2 DMEM/F12 medium in presence of 20 mM SCFAs at 37°C, 5% CO_2_ for 24 h, respectively. SCFAs mixtures contained 12 mM sodium acetate, 5 mM sodium propionate, and 3 mM sodium butyrate (Sigma-Aldrich). For pH adjustment, the medium was acidified using 1 M HCl.

### qRT-PCR

Total RNA was isolated from cultured cells using TRIzol reagent. RT was performed using an RT Kit (Takara, Code No. RR036A). The RT reaction mixtures contained 1 μg total RNA and 5× PrimeScript RT Master Mix in a final volume of 20 μL. The RT reactions were performed for 15 min at 37°C. Reverse transcriptase was inactivated by heating to 85°C for 5 s. qRT-PCR was performed with a SYBR Premix Ex Taq II Kit (Takara, Code Nos. RR820A). The qRT-PCR included an initial denaturation at 95°C for 30 s, followed by 40 cycles at 95°C for 5 s and 60°C for 30 s. All primers used are listed in Table 1. The relative expression of target genes was normalized to that of GAPDH and calculated using the 2^−ΔΔ*CT*^ method.

**Table 1:** Reverse-transcription PCR primer.

**Gene**	**Primer sequence, 5^**′**^ to 3^**′**^**	**Source**	**Size (bp)**
MCT1	F: CAATGCCACCAGCAGTTG R: GCAAGCCCAAGACCTCCAAT	NM_001037319.1	376
MCT4	F: AGCGTCTGAGCCCAGGGAGG R: ACCTCGCGGCTTGGCTTCAC	NM_001109980.2	223
NHE1	F: GAAAGACAAGCTCAACCGGTTT R: GGAGCGCTCACCGGCTAT	NM_134647.4	67
NHE2	F: TTGGAGAGTCCCTGCTGAAC R: GGCCGTGATGTAGGACAAAT	XM_002691185.5	257
NHE3	F: AGCCTTCGTGCTCCTGACA R:TGACCCCTATGGCCCTGTAC	NM_001103635.3	56
GPR41	F: AACCTCACCCTCTCGGATCT R: GCCGAGTCTTGTACCAAAGC	NM_001145233.1	214
IL1β	F: CAGTGCCTACGCACATGTCT R: AGAGGAGGTGGAGAGCCTTC	NM_174093.1	209
IL6	F: TCCTTGCTGCTTTCACACTC R: CACCCCAGGCAGACTACTTC	NM_173923.2	129
TNFα	F: GCCCTCTGGTTCAGACACTC R: AGATGAGGTAAAGCCCGTCA	NM_173966.3	192
CXCL8	F: TGGGCCACACTGTGAAAAT R: TCATGGATCTTGCTTCTCAGC	NM_173925.2	136
CCL20	F: TTCGACTGCTGTCTCCGATA R: GCACAACTTGTTTCACCCACT	NM_174263.2	172
CXCL2	F: CCCGTGGTCAACGAACTGCGCTGC R: CTAGTTTAGCATCTTATCGATGATT	NM_174299.3	204
CXCL3	F: CCCGTGGTCAACGAACTGCGCTGC R: AGTTGGTGCTGCCCTTGTTTAG	NM_001046513.2	217
CXCL5	F: TGAGACTGCTATCCAGCCG R: AGATCACTGACCGTTTTGGG	NM_174300.2	193
CXCL14	F: AAGCTGGAAATGAAGCCAAA R: GTTCCAGGCGTTGTACCATT	NM_001034410.2	153
Occludin	F: GAACGAGAAGCGACTGTATC R: CACTGCTGCTGTAATGAGG	NM_001082433.2	122
Claudin-1	F: CGTGCCTTGATGGTGAT R: CTGTGCCTCGTCGTCTT	NM_001001854.2	102
ZO-1	F: TCTGCAGCAATAAAGCAGCATTTC R: TTAGGGCACAGCATCGTATCACA	XM_010817146.1	187
GAPDH	F: GGGTCATCATCTCTGCACCT R: GGTCATAAGTCCCTCCACGA	NM_001034034.2	176

*F, forward; R, reverse*.

### Western Blotting

Cells were lysed to extract the entire protein contained in a radioimmunoprecipitation assay buffer [50 mM Tris–HCl (pH 7.4), 150 mM NaCl, 1% NP-40, 0.1% SDS] containing 1×protease inhibitor buffer. Protein concentrations were determined using a BCA kit (Thermo Scientific, Shanghai, China). Equal amounts of protein lysates were fractionated by SDS-PAGE and transferred to nitrocellulose membranes (PALL, Shanghai, China). The membranes were blocked with 5% skimmed milk and then incubated with gentle shaking overnight at 4°C, with the primary antibody plus 5% bovine serum albumin in Tris-buffered saline with Tween (TBS-T: 10 mM Tris–HCl, pH 7.5, 150 mM NaCl, 0.05% Tween 20). The following primary antibodies are β-actin, GAPDH (1: 1,000; Cell Signaling Technology, Shanghai, China), cytokeratin 18 (1: 1,000; Abcam, Shanghai, China), and SV40T (1: 100; Santa Cruze, Shanghai, China). The horseradish peroxidase (HRP)-conjugated secondary antibodies are goat anti-rabbit IgG and horse anti-mouse IgG (1: 5,000; Cell Signaling Technology). The target bands were detected using the Pierce ECL Plus Western Blotting Substrate (Thermo Scientific).

### Statistical Analysis

All data are presented as means ± standard error of the results of three independent experiments. The distribution of normality and homogeneity of variances were studied with a Kolmogorov-Smirnov and Levene test, respectively. In addition to the results of CCL20 gene expression, results of all genes expression were distributions of normality. The significance test of homogeneity of variances is <0.05 for the CXCL2 and CXCL8 gene expression. The CCL20, CXCL2, and CXCL8 gene expression (2^−ΔΔ*Ct*^) assays were log_10_ transformed for statistical analysis. Then, statistical analysis was evaluated by one-way analysis of variance (ANOVA), followed by determination of the least significant difference (LSD) for *post-hoc* multiple comparisons of treatment means and tested by the independent sample *t*-test (qRT-PCR from Table 2), using SPSS 19.0 software (SPSS Inc.; Chicago, IL, USA). The cluster was analyzed with a PlotHeatMap function by R programming language. Correlation analysis was performed using GraphPad Prism version 6.00 (GraphPad Software, San Diego, CA; www.graphpad.com) to verify whether there were possible relationships between the GPR41 mRNA expression and chemokines mRNA expression. *P* values < 0.05 were considered significant.

**Table 2:** Comparisons between RNA-seq and qRT-PCR results.

	**Treatment**^1^					
**Gene symbol**	**RNA-seq**	**SEM**	***P*-value**	**qRT-PCR**	**SEM**	***P-*value**
IL1β	4.8	0.62	<0.001	13.17	1.75	0.006
IL6	0.6	0.27	0.01	0.69	1.38	0.08
TNFα	10.82	0.47	<0.001	38.11	2.73	<0.001
CCL20	15.87	0.33	<0.001	20.63	0.83	<0.001
CXCL2	239.85	0.33	<0.001	42.59	2.15	<0.001
CXCL3	16.59	0.24	<0.001	24.02	1.38	<0.001
CXCL5	9.34	0.07	<0.001	14.43	1.3	<0.001
CXCL8	8.28	0.11	<0.001	11.51	0.27	<0.001
CXCL14	9.59	0.69	<0.001	47.9	7.97	0.001
GPR41	11.52	0.68	<0.001	5.27	1.19	0.02

1 *Correspond to the fold change (2-ΔΔCT) of 20 mM SCFAs vs. control. Fold change (> 1) is upregulated and fold change (< 1) is downregulated in BRECs incubated with 20 mM SCFAs compared with control BRECs. CT, quantification cycle*.

## Results

### Establishment and Characterization of Immortal Bovine Rumen Epithelial Cells

**Figure 1:**
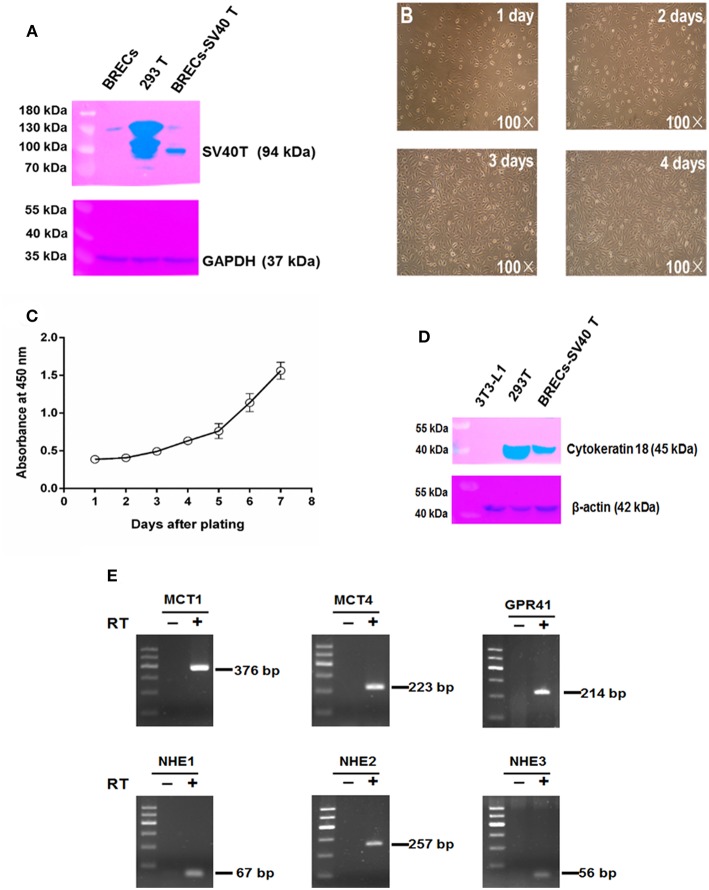
Characterization of immortal bovine rumen epithelial cells (BRECs). **(A)** Immunoblotting of SV40T. BRECs: not infected, as negative control, 293T: as positive control, BRECs-SV40T: the infected cells used in the present study. **(B)** Morphology of immortal BRECs at 1~4 days of culture 100×. **(C)** Growth curve of immortal BRECs at 1~7 days of culture. **(D)** Immunoblotting of cytokeratin 18. 3T3-L1 fibroblast, as negative control, 293T: as positive control, BRECs-SV40T: the infected cells used in the present study. **(E)** RT-PCR analyses of MCT1, MCT4, NHE1, NHE2, NHE3, and GPR41. DNA Marker: 600, 500, 400, 300, 200, and 100 bp. The reverse transcription without reverse transcriptase (RT-, negative control). Data shown are means ± SEM of three independent experiments (*n*= 3).

To overcome primary BRECs senescence, primary BRECs were immortalized using SV40T. An immunoblotting band was observed for SV40T (Figure 1A). When the immortal BRECs were seeded at 2 × 10^5^ cells/ 25 cm^2^, these cells reached 100% confluence after 4 days of culture (Figure 1B). In addition, the immortal BRECs maintained typical epithelial-like and “cobblestone” morphology (Figure 1B). The immortal BRECs maintained the ability to proliferate robustly without showing any signs of senescence (Figure 1C) and cultured passages 50 at least. An immunoblotting band was found for cytokeratin 18 in immortal BRECs and 293T positive cells, but not in 3T3-L1 fibroblast (Figure 1D). The result indicates that immortal BRECs originated from epithelial cells type. To further assess the expression of gene involved in SCFAs transporters and receptors in immortal BRECs such as MCT1, MCT4, NHE1, NHE2, NHE3, and GPR41, these genes were analyzed using RT-PCR. RT-PCR analyses confirmed the expression of MCT1, MCT4, NHE1, NHE2, NHE3, and GPR41 in immortal BRECs (Figure 1E). These results indicate that BRECs was derived from rumen cells of epithelial origin, and that they may transport and absorb SCFAs.

### Genes Differentially Expressed in BRECs After the 20 mM SCFAs Treatment

**Figure 2:**
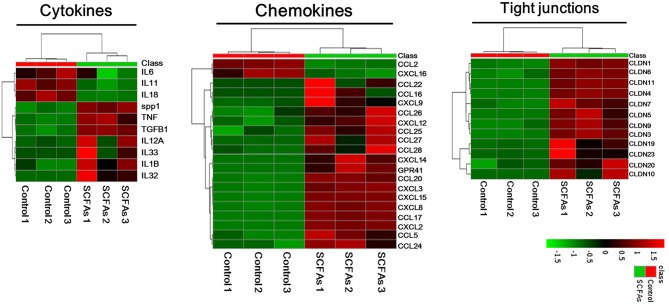
Genes differentially expressed between BRECs without SCFAs and BRECs with SCFAs treatment. The BRECs were cultured in the absence (controls) or presence (treatment groups) of 20 mM SCFA for 24 h. The 20 mM SCFA mixture consisted of 12 mM sodium acetate, 5 mM sodium propionate, and 3 mM sodium butyrate. Treeviews of selected cytokines, chemokines, and TJ genes up-, or down-regulated after 20 mM SCFAs treatment. Data shown are based on three independent experiments (*n* = 3).

To understand the new insights into cell responses regulated by SCFAs and GPR41 in BRECs, transcriptome analysis was performed. Transcriptome analysis revealed a marked difference in gene expression profile between the BRECs and BRECs induced by 20 mM SCFAs (Figure 2). The proinflammatory cytokines TNFα and IL1β were markedly enhanced by the induction of 20 mM SCFAs (*p* < 0.01). Interestingly, the proinflammatory cytokines IL6 was attenuated, although the difference was not significant compared with the control group (*p* > 0.05). Many genes involved in CCL and CXCL chemokines were significantly enhanced (*p* < 0.01). The BRECs induced by 20 mM SCFAs showed profound changes in many genes involved in TJ (*p* < 0.01). In addition, the expression of proinflammatory cytokines, chemokines, and GPR41 were confirmed with qRT-PCR, which is consistent with the analysis of gene expression profiling (Table 2). These results indicate that SCFAs may regulate the genes expressions of proinflammatory cytokine, chemokines, and TJ via GPR41 in BRECs.

### Establishment of GPR41 Knockdown BRECs Cell Lines

**Figure 3:**
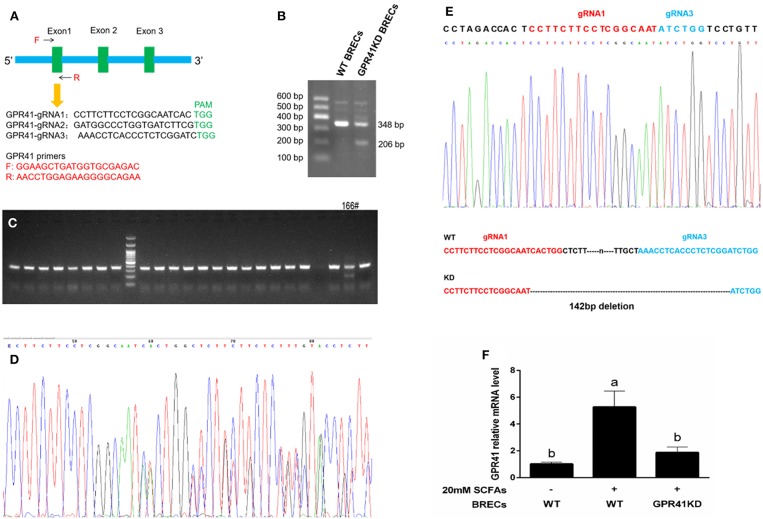
Generation of GPR41-knocdown (GPR41KD) BRECs cell lines using the CRISPR/Cas9 system. **(A)** Schematic representation of the GPR41 target gRNA sequences. Arrows represent primer positions. PAM, protospacer adjacent motif. GPR41 primer is used to amplify genomic DNA sequence range between GPR41-gRNA 1 and GPR41-gRNA3. **(B)** The target GPR41 genomic region PCR products. **(C)** The screening positive target by Cruiser™ Enzyme. Amplicons digested by Cruiser™ Enzyme were separated in 2% agarose gel electrophoresis. **(D)** The sequence analysis of positive target. **(E)** The sequence analysis of TA clone. The deleted sequences in GPR41-knocdown BRECs cell lines are presented. **(F)** qRT-PCR analysis of GPR41 level in the GPR41-knocdown BRECs. WT, Wild type.

To evaluate the functions of GPR41 gene, we generated GPR41KD BRECs cell lines using CRISPR/Cas9 system. We firstly designed three gRNAs against exon 1 of GPR41 to edit GPR41 exon 1 by CRISPR/Cas9 system (Figure 3A). The targeted GPR41 genomic region PCR products amplified by GPR41 primers from clone cell were shown (Figure 3B). The targeted GPR41 genomic region PCR products were incubated using Cruiser™ Enzyme. The result showed that two bands were found in 166# (Figure 3C). Next, the PCR products performed the sequencing analysis. Sequence analysis of the PCR products revealed the DNA peak (Figure 3D). The result indicate that at GPR41, one allele had a deletion between the gRNA1 and gRNA3 regions; however, another allele was not edited. To determine the exact target site of GPR41 deletion, the targeted GPR41 genomic region PCR products were cloned into the T vector to analyze the target PCR products sequence. Sequence analysis of the T vector revealed that GPR41 had 142 bp deletion between gRNA1 and gRNA3 region (Figure 3E). qRT-PCR analyses confirmed the expression of GPR41 in GPR41KD BRECs (Figure 3F). Therefore, GPR41KD BRECs cell lines was established, not knockout.

### Regulation of the Immune Response via GPR41

**Figure 4:**
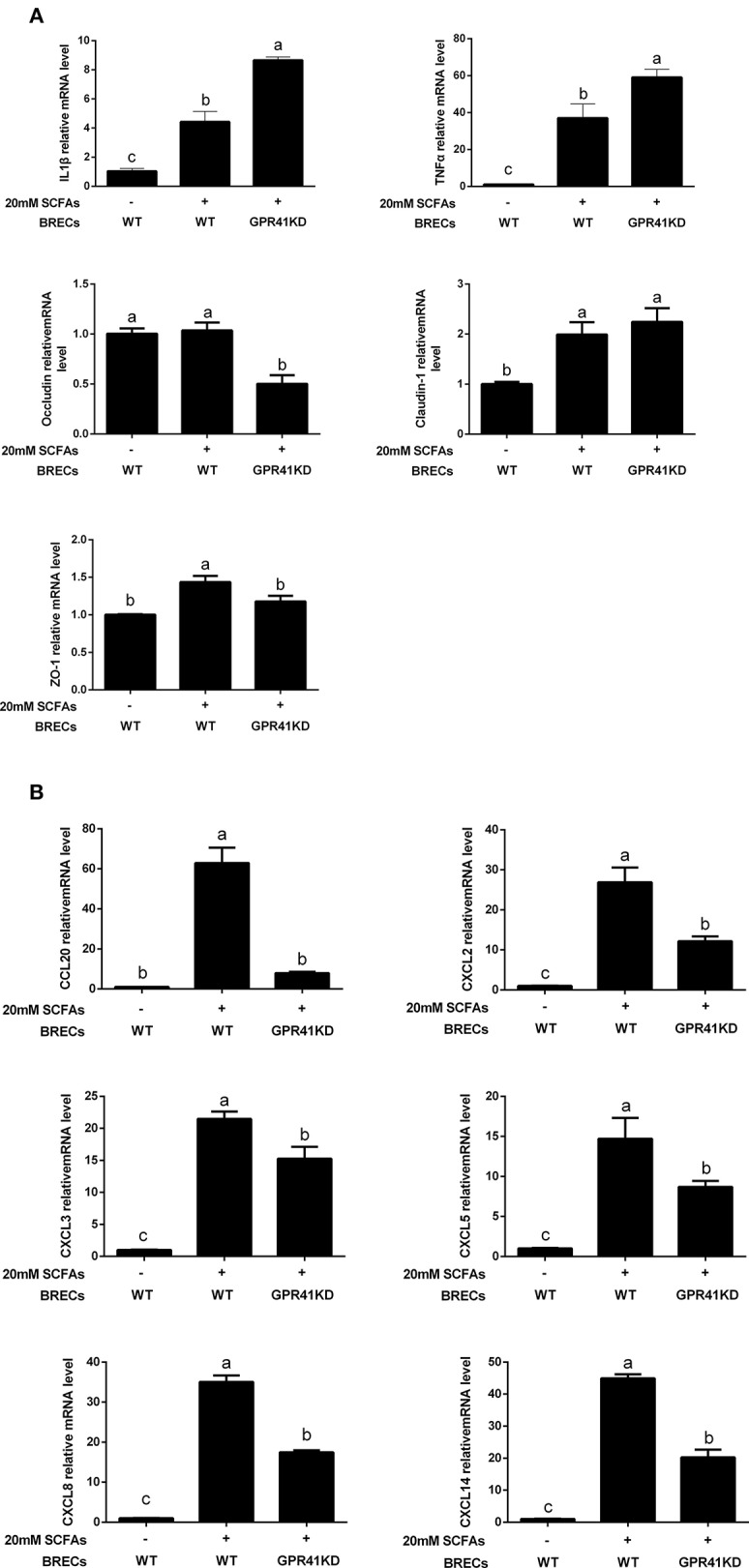
Quantitative real-time polymerase chain reaction analysis of selected genes. All of the data were obtained at 24 h from untreated BRECs or 20 mM SCFAs treated BRECs or 20 mM SCFAs treated GPR41KD BRECs. qRT-PCR analysis of IL1β, TNFα, occludin, claudin-1, ZO-1 **(A)**, CCL20, CXCL2, CXCL3, CXCL5, CXCL8, and CXCL14 **(B)** expression levels. GAPDH was used as an internal reference gene. Different lowercase letters indicate significant differences. Data shown are means ± SEM of three independent experiments (*n* = 3). WT, Wild type.

The GPR41KD BRECs with 20 mM SCFAs treatment significantly enhanced the proinflammatory cytokines expression of IL1β and TNFα compared with BRECs with 20 mM SCFAs treatment (Figure 4A; *p* < 0.05). In the contrary, the expression of occludin and ZO-1 genes involved in immune barrier were markedly downregulated in GPR41KD BRECs treated with 20 mM SCFAs, relative to BRECs treated with 20 mM SCFAs (Figure 4A; *p* < 0.05). In addition, CXCL2, CXCL3, CXCL5, CXCL8, and CXCL14 were significantly attenuated in GPR41KD BRECs treated with 20 mM SCFAs compared with BRECs with 20 mM SCFAs treatment (Figure 4B; *p* < 0.05). These results revealed that SCFAs regulate the immune responses by GPR41 in BRECs.

### Correlations Between GPR41 and Chemokines, TJ Proteins, and Proinflammatory Cytokine

**Figure 5:**
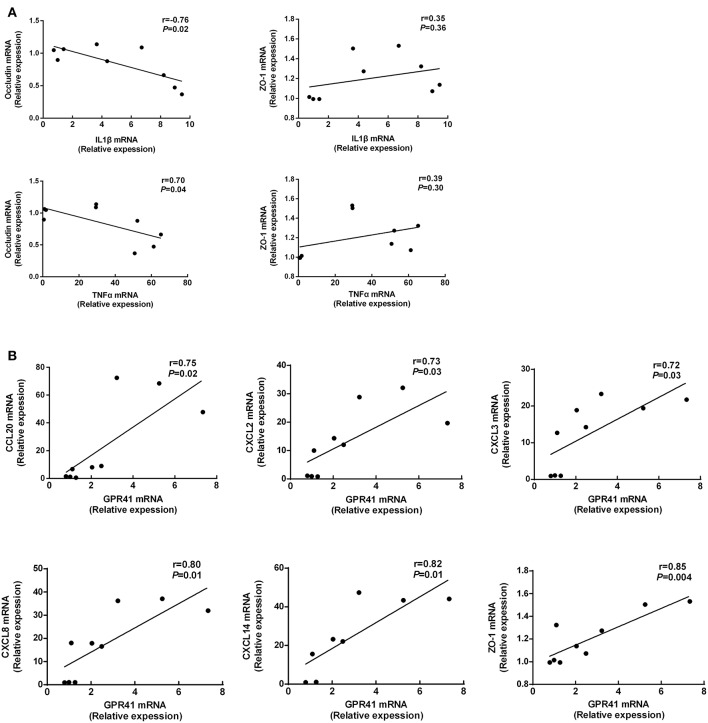
Correlations between GPR41 mRNA expression and chemokines, TJ proteins mRNA expression **(A,B)**. The figure includes data obtained at 24 h from untreated BRECs, 20 mM SCFAs treated BRECs, or 20 mM SCFAs treated GPR41KD BRECs. Data shown are means ± SEM of three independent experiments (*n* = 3).

The relationships between proinflammatory cytokine and TJ protein expression is shown (Figure 5A). There is a negative correlation between occluding mRNA expression and IL1β mRNA expression (Figure 5A; *p* < 0.05). In addition, occluding mRNA expression is a negative correlation with TNFα mRNA expression (Figure 5A; *p* < 0.05). These results suggest that mRNA expression of occluding is involved in the mRNA expression of the proinflammatory cytokine IL1β and TNFα due to defective receptor for SCFAs signaling. Additionally, the GPR41 mRNA expression is positively correlated with CCL20, CXCL2, CXCL3, CXCL8, CXCL14, and ZO-1 (Figure 5B; *p* < 0.05). These data show the positive effect of GPR41 on protective immunity and epithelial immunity barrier in BRECs.

## Discussion

In the present study, immortalized BRECs were firstly established and validated for application in the investigation of the regulatory effects of SCFAs and GPR41 in immune responses. The BRECs are key immune barriers for preventing the translocation of LPS and the lysis of dead bacterial cells into blood, whereas primary BRECs are not an ideal model for long-culture owing to large amounts of programmed cell death that occurs after cells are isolated from the bovine tissues (22, 23). According to our current literature, the immortal BRECs remain not reported on. To avoid induction of programmed cell death *in vitro*, the immortalized BRECs lines were established by SV40T-induced immortalization. These cells have cultured at least 50 passages without showing any signs of senescence. To determine the origin of the immortal BRECs, we firstly performed immunoblotting analysis of cytokeratin 18. The cytokeratin 18 was found in BRECs, which is often used to distinguish cells of epithelial origin from stellate cells (24). The result demonstrated that immortal BRECs originated from epithelial-type cells. To validate whether isolated BRECs are from the rumen epithelial cells, we detected the marker genes involved SCFAs transport and uptake, such as, MCT1, MCT4, NHE1, NHE2, and NHE3. MCT1 plays a key role for direct export of SCFA (14, 25), which may be a critical rate-limiting step for acidosis protection (26). The ruminal epithelium can decrease pH acidification to maintain the pH homeostasis of rumen via Na^+^/H^+^ exchangers (27). Our result confirmed that the BRECs line expressed the MCT1, MCT4, NHE1, NHE2, and NHE3. Therefore, the immortal BRECs were characterized by the physiological function of rumen epithelium and can be used to study the regulation of immune responses by SCFAs and GPR41 in BRECs.

It has been known that SCFAs produced by rumen microbial fermentation are directly absorbed across the rumen epithelium (27). However, the bovine rumen immune system often struggles to challenge antigens from lysis of dead microorganism cells in the rumen (1–3), but can actively respond to the infection by the lysis of dead bacterial cells or free LPS from the shedding gram-negative bacteria in rumen. SCFAs involved in immune regulatory metabolites can activate the GPR41 receptor. We hypothesized that SCFAs may regulate the immune responses by GPR41 in BRECs. In this study, we have demonstrated that SCFAs activate the GPR41 to enhance the expression of genes related to the diverse chemokines and the epithelial immune barrier in BRECs. These results may provide new insights into the role of GPR41 in mediating protective immunity in ruminal epithelium tissues.

To understand the regulatory effects of SCFAs and GPR41 on innate immunity responses in BRECs, we firstly obtained the difference of mRNA in BRECs with or without 20 mM SCFAs by transcriptome analysis. The genes involved in proinflammatory cytokines (IL1β and TNFα) and immune barriers were significantly upregulated in BRECs treated with 20 mM SCFAs. Remarkably, many genes related to immune cell recruitment were also markedly enhanced by addition of 20 mM SCFAs. In addition, GPR41 was also significantly activated by SCFAs. These results suggest that upregulation of chemokines expression and immune barrier molecules may be mediated via the activation of GPR41.

To identify that GPR41 was the relevant receptor for SCFAs regulatory effects on the innate immune responses, we sourced GPR41KD BRECs by CRISPR/Cas9 system. The GPR41KD BRECs treated with 20 mM SCFAs significantly increased the levels of IL1β and TNFα, showing a proinflammatory response compared with BRECs treated with 20 mM SCFAs, but decreased the expression of genes involved in the immune barrier. Moreover, mRNA expression of occluding is negatively correlated with the TNFα mRNA expression. These results indicate that both impairment of rumen epithelial barrier and proinflammatory response can occur if GPR41 are deficient in BRECs. Once the impaired rumen epithelial permeability is increased, the LPS and the bacterial lysate have the opportunity to transfer into the bloodstream, leading to systemic inflammatory response (28). It seems to suggest that GPR41 plays a critical role in mediating protective immunity in BRECs. One study reported that GPR41-deficient mice lead to a decrease in proinflammatory cytokines IL1β, IL6, and IL11, and suggested that mice lacking of GPR41 are deficient in enhancing expression of genes involved in key inflammatory mediators in the intestine (12). The different experimental results are likely dependent on the experimental models and specific features of the cell type.

It has been shown that polymorphonuclear leukocytes build up a first line of epithelium defense. These immune cells migrate to the inflamed epithelium tissues infected with pathogens by the various chemotactic factor exerting an important role in the clearance of pathogens (5). Our results show that GPR41KD BRECs treated with 20 mM SCFAs significantly reduced the levels of CCL20, CXCL2, CXCL3, CXCL5, CXCL8, and CXCL14 compared with BRECs treated with 20 mM SCFAs. Consistently, mRNA expression of the GPR41 is positively correlated with mRNA expression of CCL20, CXCL2, CXCL3, CXCL8, and CXCL14 by correlations analysis. These results indicate that stimulation of GPR41 by SCFAs may play a critical role in clearance of LPS produced by the shedding gram-negative bacteria within rumen, because GPR41-deficient BRECs showed a decrease in expression level of chemotactic molecular and immune barrier. Consistently, the intestine tissues decreased the expression of genes involved in CCL and CXCL chemokines in the mice lacking GPR41, leading to the abnormal decrease in neutrophil infiltration (12). In fact, our study found that SCFAs enhance GPR41-mediated the expression of diverse chemokines in BRECs, and indicating that GPR41 may promote the expression of a variety of chemotactic molecules to recruit the polymorphonuclear leukocyte from rumen lamina propria to rumen epithelium. Therefore, we will focus on whether the rumen epithelium tissues exist in the immune cells in a subsequent study. In conclusion, GPR41 as receptors for SCFAs potentially provides a molecular link between diets and immune responses in ruminants, and SCFAs enhance GPR41-mediated the expression of genes related to polymorphonuclear leukocyte recruitment and epithelial barrier function and thereby mediate protective immunity in BRECs.

## Ethics Statement

This study was carried out in accordance with the principles of Yang Zhou University, the Institutional Animal Care and Use Committee. The protocol was approved by the Institutional Animal Care and Use Committee.

## Author Contributions

KZ performed the experiments, analyzed the data, and wrote the manuscript. XG, MJ, and TY also performed the experiments work. YC revised the manuscript. GZ contributed to the experimental idea.

### Conflict of Interest Statement

The authors declare that the research was conducted in the absence of any commercial or financial relationships that could be construed as a potential conflict of interest.
